# Supplementary figures and images for: Antimicrobial drug resistant features of Mycobacterium tuberculosis associated with treatment failure
Source: PLoS One. 2023 Oct 26;18(10):e0293194. doi: 10.1371/journal.pone.0293194 (PMC10602240; doi:10.1371/journal.pone.0293194)

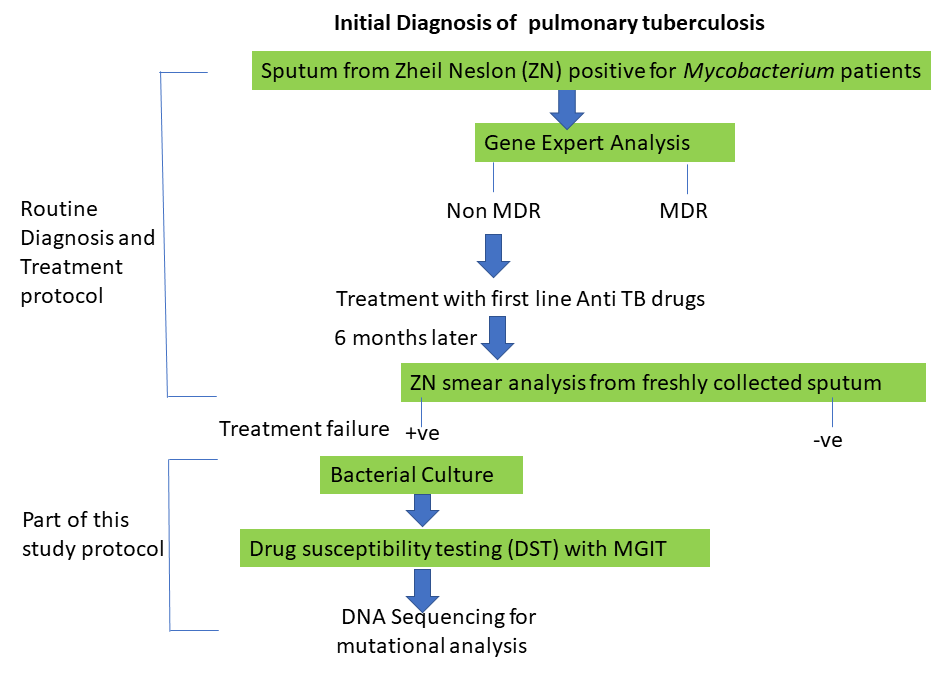

Supplement: S1 Fig — (TIF) [file pone.0293194.s001.TIF]
