# Supplementary material for: Antimicrobial drug resistant features of Mycobacterium tuberculosis associated with treatment failure
Source: PLoS One. 2023 Oct 26;18(10):e0293194. doi: 10.1371/journal.pone.0293194 (PMC10602240; doi:10.1371/journal.pone.0293194)
Supplement: S1 File — (DOCX) [file pone.0293194.s002.docx]

Supplementary Information

Antimicrobial drug resistant features of *Mycobacterium tuberculosis* associated with treatment failure in Pakistan

Fizza Mushtaq^1^, Syed Mohsin Raza^1^, Adeel Ahmad^2^, Hina Aslam^1^, Atiqa Adeel^2^, Sidrah Saleem^2^, Irfan Ahmad^1,3*^

^1^Institute of Biomedical and Allied Health Sciences, University of Health Sciences, Lahore, Pakistan

^2^Department of Microbiology, University of Health Sciences, Lahore, Pakistan

^3^Department of Molecular Biology and Umeå Centre for Microbial Research (UCMR), Umeå University, Umeå, Sweden

*Correspondence: irfan.ahmad@umu.se

**Table S1: Primers Used in the study**

| **Sr No** | **Primers** | **5’-3’ Sequence** | **Source** |
| --- | --- | --- | --- |
| **Strain typing primers** | | |  |
| 1 | F-16S rRNA gene  R-16S rRNA gene | 5’ GAGATACTCGAGTGGCGAAC 3´  5`CAACGCGACAAACCACCTAC3´ | ^1^ |
| 2 | *F-rv0577*  R-rv0577 | 5` ATGCCCAAGAGAAGCGAATACA 3´  5` AATGTCAGCCGGTTCCGCAA 3´ | ^1^ |
| 3 | F-RD9  R-RD9 | 5` GTGTAGGTCAGCCCCATCC 3´  5` GTAAGCGCGTGGTGTGGA 3´ | ^1^ |
| 4 | *F-mtbk_20680*  R-mtbk_20680 | 5` TTATGCCAGAAATACACCCGCG 3´  5` AATCGCGGGCTTGTGGCTAC 3´ | ^1^ |
| 5 | F-IS*1311*  R-IS1311 | 5` TCGATCAGTGCTTGTTCGCG 3´  5` CGATGGTGTCGAGTTGCTCT 3´ | ^1^ |
| 6 | F-DT1  R-DT1 | 5` AAGGTGAGCCCAGCTTTGAACTCCA 3´  5` GCGCTTCATTCGCGATCATCAGGTG 3´ | ^1^ |
| 7 | *F-mass_3210*  R-mass_3210 | 5` GCTTGTTCCCGGTGCCACAC 3´  5` GGAGCGCGATGCGTCAGGAC 3´ | ^1^ |
| 8 | *F-mkan_rs12360*  R-mkan_rs12360 | 5` ACAAACGGTGTGTCGCAATGTGCCA 3´  5` TGTCGAGCAGACGTTCCAGGACGGT 3´ | ^1^ |
| 9 | F-RD750  R-RD750 | 5` TTGCACAGCTTGGCGACGAAT 3´  5` ATGGCCTCGTGTCTCCCAAAACT 3´ | ^1^ |
| Sequencing  primers | | |  |
| 10 | *rpoB* (F)  *rpoB* (R) | 5′-TGCACGTCGCGGACCTCCA-3′  5′-TCGCCGCGATCAAGGAGT-3′ | This study |
| 11 | *katG* (F)  *katG* (R) | 5′-TGGCCGCGGCGGTCGACATT-3′  5′-GGTCAGTGGCCAGCATCGTC-3′ | This study |
| 12 | *wib7* (F)  *wib7* (R) | 5′-GCTGGTTCGCGGTCGGACCT-3′  5′-CGGGGTATCGGCGAACCACA-3′ | This study |
| 13 | *eis* (F)  *eis* (R) | 5′-GCCAGACACTGTCGTCGTAA-3′  5′-CGAAAAGCCCGTCAGCCTA-3′ | This study |
| 14 | *rrs* (F)  *rrs* (R) | 5′-CGGATTGACGGTAGGTGGAG-3′  5′-CCTACGAGCTCTTTACGCCC-3′ | This study |
| 15 | *pncA (F)*  *pncA (R)* | 5′-GCGTCATGGACCCTATATC-3′  5′-AACAGTTCATCCCGGTTC-3′ | This study |
| RT-PCR  primers | | |  |
| 16 | *F- Rv1258C*  *F- Rv1258C* | F-CCTCAACCTGGCCTTTATTG  R-GGATGGACAACCCGAATG | This study |
|  | *F-polA*  *R- polA* | F-GTCGTGGTTGGACCTTGGAGGG  R-GCGTCCGTATCGTCGTCATCG | This study |

| **Strain ID**  **Table S2 PCR based screening of isolates for the presence of *Mycobacterium tuberculosis* specific gene loci** | Type based on drug susceptibility | 16s RNA gene | Rv0577 | RD9 | Mtbk-20680 | RD750 |
| --- | --- | --- | --- | --- | --- | --- |
| MTBLH001 | MDR | + | + | + | - | + |
| MTBLH002 | Mono-DR | + | - | + | + | - |
| MTBLH003 | MonoDR | + | - | + | + | - |
| MTBLH004 | Non-MDR | + | - | + | + | - |
| MTBLH005 | Non-MDR | + | - | + | + | - |
| MTBLH006 | Non-MDR | + | - | + | + | - |
| MTBLH007 | MDR | + | - | + | + | - |
| MTBLH008 | MDR | + | - | + | + | - |
| MTBLH009 | Non-MDR | + | - | - | - | - |
| MTBLH010 | MDR | + | - | + | + | - |
| MTBLH011 | Non-MDR | + | - | + | + | - |
| MTBLH012 | Non-MDR | + | - | + | + | - |
| MTBLH013 | Mono-DR | + | + | + | - | + |
| MTBLH014 | Non-MDR | + | - | + | + | - |
| MTBLH015 | Non-MDR | + | - | + | + | - |
| MTBLH016 | MDR | + | - | + | + | - |
| MTBLH017 | Mono-DR | + | + | + | + | + |
| MTBLH018 | MDR | + | - | - | + | - |
| MTBLH019 | Non-MDR | + | - | - | - | - |
| MTBLH020 | Mono-DR | + | + | + | + | - |
| MTBLH021 | MDR | + | + | + | - | + |
| MTBLH022 | MDR | + | - | + | + | - |
| MTBLH023 | MDR | + | - | + | + | - |
| MTBLH024 | MDR | + | - | + | + | - |
| MTBLH025 | MDR | + | + | + | + | - |
| MTBLH026 | MDR | + | + | + | + | - |
| MTBLH027 | MDR | + | - | + | - | + |
| MTBLH028 | MDR | + | - | + | + | - |
| MTBLH029 | MDR | + | + | + | + | - |
| MTBLH030 | MDR | + | - | + | + | - |
| MTBLH031 | MDR | + | + | + | - | + |
| MTBLH032 | MDR | + | - | + | + | - |
| MTBLH033 | MDR | + | + | + | + | - |
| MTBLH034 | MDR | + |  |  |  |  |
| MTBLH035 | MDR | + | - | + | + | + |
| MTBLH036 | Non-MDR | + | + | + | + | -, |
| MTBLH037 | MDR | + | - | + | + | - |
| MTBLH038 | MDR | + | - | + | + | - |
| MTBLH039 | MDR | + | - | + | + | - |
| MTBLH040 | MDR | + | - | + | + | + |
| MTBLH041 | MDR | + | - | + | + | - |
| MTBLH042 | MDR | + | - | + | + | - |
| MTBLH043 | MDR | + | - | - | + | - |
| MTBLH044 | MDR | + | - | + | + | - |
| MTBLH045 | MDR | + | - | + | + | - |
| MTBLH046 | MDR | + | - | + | + | - |
| MTBLH047 | MDR | + | + | + | - | + |
| MTBLH048 | MDR | + | + | + | - | + |
| MTBLH049 | Non-MDR | + | - | + | + | - |
| MTBLH050 | MDR | + | + | + | - | + |
| MTBLH051 | MDR | + | - | + | + | - |
| MTBLH052 | MDR | + | - | - | - | - |
| MTBLH053 | MDR | + | + | + | - | + |
| MTBLH054 | MDR | + | + | + | - | + |
| MTBLH055 | MDR | + | - | + | + | - |
| MTBLH056 | MDR | + | - | - | - | - |
| MTBLH057 | MDR | + | - | + | + | - |
| MTBLH058 | MDR | + | - | + | + | - |
| MTBLH059 | MDR | + | + | + | - | + |
| MTBLH060 | MDR | + | + | + | - | + |
| MTBLH061 | MDR | + | + | + | - | + |
| MTBLH062 | MDR | + | - | + | + | - |
| MTBLH063 | MDR | + | - | - | - | - |
| MTBLH064 | MDR | + | - | + | + | - |
| MTBLH065 | Non-MDR | + | - | + | + | - |
| MTBLH066 | Non-MDR | + | - | - | - | - |
| MTBLH067 | Non-MDR | + |  |  |  |  |
| MTBLH068 | Non-MDR | + | - | + | + | - |
| MTBLH069 | MonoDR | + | - | + | + | - |
| MTBLH070 | Non-MDR | - | - | - | + | - |
| MTBLH071 | Non-MDR | - | - | - | - | - |
| MTBLH072 | 66M | - | - | - | - | - |
| MTBLH073 | 21G | + | - | + | + | - |
| MTBLH074 | 22G | + | - | + | + | - |
| MTBLH075 | 23G | + | - | + | + | - |
| MTBLH076 | 31M | + | - | + | + | - |
| MTBLH077 | Non-MDR | + | - | + | + | - |
| MTBLH078 | Non-MDR | + | - | + | + | - |
| MTBLH079 | Non-MDR | + | - | + | + | - |
| MTBLH080 | Non-MDR | + | - | + | + | - |
| MTBLH081 | Non-MDR | + | - | - | - | - |
| MTBLH082 | Non-MDR | + | - | + | + | - |
| MTBLH083 | Non-MDR | + | - | + | + | - |
| MTBLH084 | Non-MDR | + | - | + | + | - |
| MTBLH085 | Non-MDR | + | + | + | - | + |
| MTBLH086 | Non-MDR | + | - | + | + | - |
| MTBLH087 | Non-MDR | + | - | + | + | - |
| MTBLH088 | Non-MDR | + | + | + | + | -, |
| MTBLH089 | Non-MDR | + | - | + | + | - |
| MTBLH090 | Non-MDR | + | - | + | + | - |
| MTBLH091 | Non-MDR | + | - | + | + | - |
| MTBLH092 | Non-MDR | + | - | + | + | - |
| MTBLH093 | Non-MDR | + | - | - | - | - |
| MTBLH094 | Non-MDR | + | - | + | + | - |
| MTBLH095 | Non-MDR | + | - | + | + | - |
| MTBLH096 | Non-MDR | + | - | + | + | - |
| MTBLH097 | MonoMDR | + | + | + | - | + |
| MTBLH098 | Non-MDR | + | - | + | + | - |
| MTBLH099 | Non-MDR | + | - | + | + | - |
| MTBLH100 | Non-MDR | + | + | + | + | -, |
| MTBLH101 | Non-MDR | + | - | + | + | - |
| MTBLH102 | Non-MDR | + | - | - | - | - |
| MTBLH103 | Non-MDR | + | - | + | + | - |
| MTBLH104 | Non-MDR | + | - | + | + | - |
| MTBLH105 | Non-MDR | + | - | + | + | - |
| MTBLH106 | Non-MDR | + | + | + | - | + |
| MTBLH107 | Non-MDR | + | - | + | + | - |
| MTBLH108 | Non-MDR | + | - | - | - | - |
| MTBLH109 | Non-MDR | + | - | + | + | - |
| MTBLH110 | Non-MDR | + | - | + | + | - |
| MTBLH111 | Non-MDR | + | - | + | + | - |
| MTBLH112 | Non-MDR | + | + | + | - | + |
| MTBLH113 | Non-MDR | + | - | + | + | - |
| MTBLH114 | Non-MDR | + | + | + | + | -, |
| MTBLH115 | Non-MDR | + | - | + | + | - |
| MTBLH116 | Non-MDR | + | - | - | - | - |
| MTBLH117 | Non-MDR | + | - | + | + | - |
| MTBLH118 | Non-MDR | + | - | + | + | - |
| MTBLH119 | Non-MDR | + | - | + | + | - |
| MTBLH120 | Non-MDR | + | + | + | - | + |
| MTBLH121 | Non-MDR | + | - | + | + | - |
| MTBLH122 | Non-MDR | - | - | - | - | - |
| MTBLH123 | Non-MDR | + | - | + | + | - |
| MTBLH124 | Non-MDR | + | - | + | + | - |
| MTBLH125 | Non-MDR | + | - | + | + | - |
| MTBLH126 | Non-MDR | - | - | - | - | - |
| MTBLH12 | Non-MDR | + | - | + | + | - |
| MTBLH128 | Non-MDR | + | - | + | + | - |

**Table 3: Source, year of isolation and drug susceptibility pattern of *M. tuberculosis* isolates used in the study**

| **Strain ID** | **Place of isolation** | **Year of isolation** | **Drug resistance pattern** | **Drug ssusceptibility pattern** |
| --- | --- | --- | --- | --- |
| MTBLH001 | Mayo Hospital | 2016 | RIF, INH | AK, PYZ, LZD, MOX |
| MTBLH002 | Mayo Hospital | 2016 | AK | INH, RIF, PYZ, LZD, MOX |
| MTBLH003 | Mayo Hospital | 2016 | PYZ | INH, RIF, AK LZD, MOX |
| MTBLH004 | Mayo Hospital | 2016 | - | INH, RIF, AK, PYZ, LZD, MOX |
| MTBLH005 | Mayo Hospital | 2016 | . | INH, RIF, AK, PYZ, LZD, MOX |
| MTBLH006 | Mayo Hospital | 2016 | - | INH, RIF, AK, PYZ, LZD, MOX |
| MTBLH007 | Mayo Hospital | 2016 | RIF, INH | AK, PYZ, LZD, MOX |
| MTBLH008 | Mayo Hospital | 2016 | AK, PYZ | RIF, INH, LZD, MOX |
| MTBLH009 | Mayo Hospital | 2016 | - | INH, RIF, AK, PYZ, LZD, MOX |
| MTBLH010 | Mayo Hospital | 2016 | RIF, INH, AK | PYZ, LZD, MOX |
| MTBLH011 | Mayo Hospital | 2016 | - | INH, RIF, AK, PYZ, LZD, MOX |
| MTBLH012 | Mayo Hospital | 2016 | - | INH, RIF, AK, PYZ, LZD, MOX |
| MTBLH013 | Mayo Hospital | 2016 | PYZ | INH, RIF, AK, LZD, MOX |
| MTBLH014 | Mayo Hospital | 2016 | - | INH, RIF, AK, PYZ, LZD, MOX |
| MTBLH015 | Mayo Hospital | 2016 |  | INH, RIF, AK, PYZ, LZD, MOX |
| MTBLH016 | Mayo Hospital | 2016 | INH, RIF | AK, PYZ, LZD, MOX |
| MTBLH017 | Mayo Hospital | 2016 | PYZ | INH, RIF, AK, LZD, MOX |
| MTBLH018 | Mayo Hospital | 2016 | INH, RIF, AK, PYZ | LZD, MOX |
| MTBLH019 | Mayo Hospital | 2016 | - | INH, RIF, AK, PYZ, LZD, MOX |
| MTBLH020 | Gulab Devi Hospital | 2016 | PYZ | INH, RIF, AK, LZD, MOX |
| MTBLH021 | Gulab Devi Hospital | 2016 | RIF, INH,PYZ | AK, LZD, MOX |
| MTBLH022 | Gulab Devi Hospital | 2016 | RIF, INH,PYZ | AK, LZD, MOX |
| MTBLH023 | Gulab Devi Hospital | 2016 | RIF, INH, AK | PYZ, LZD, MOX |
| MTBLH024 | Gulab Devi Hospital | 2016 | RIF, INH | AK, PYZ, LZD, MOX |
| MTBLH025 | Gulab Devi Hospital | 2016 | RIF, INH,PYZ | AK, LZD, MOX |
| MTBLH026 | Gulab Devi Hospital | 2016 | RIF, INH,AK, PYZ | LZD, MOX |
| MTBLH027 | Gulab Devi Hospital | 2016 | RIF, INH,AK, PYZ | LZD, MOX |
| MTBLH028 | Gulab Devi Hospital | 2016 | RIF, INH,AK, PYZ | LZD, MOX |
| MTBLH029 | Gulab Devi Hospital | 2016 | RIF, INH, PYZ | AK, LZD, MOX |
| MTBLH030 | Gulab Devi Hospital | 2016 | RIF, INH, PYZ | AK, LZD, MOX |
| MTBLH031 | Gulab Devi Hospital | 2016 | RIF, INH,AK, PYZ | LZD, MOX |
| MTBLH032 | Gulab Devi Hospital | 2016 | RIF, INH, PYZ | AK, LZD, MOX |
| MTBLH033 | Gulab Devi Hospital | 2016 | RIF, INH, PYZ | AK, LZD, MOX |
| MTBLH034 | Gulab Devi Hospital | 2016 | RIF, INH, PYZ | AK, LZD, MOX |
| MTBLH035 | Gulab Devi Hospital | 2016 | RIF, INH | AK, PYZ, LZD, MOX |
| MTBLH036 | Gulab Devi Hospital | 2016 | - | RIF, INH, AK, LZD, MOX |
| MTBLH037 | Gulab Devi Hospital | 2016 | RIF, INH, PYZ | AK, LZD, MOX |
| MTBLH038 | Gulab Devi Hospital | 2016 | RIF, INH, AK, PYZ | MOX, LZD |
| MTBLH039 | Gulab Devi Hospital | 2016 | RIF, INH | AK, PYZ, LZD, MOX |
| MTBLH040 | Gulab Devi Hospital | 2016 | RIF, INH, PYZ | AK, PYZ, LZD, MOX |
| MTBLH041 | Gulab Devi Hospital | 2016 | RIF, INH | AK, PYZ, LZD, MOX |
| MTBLH042 | Gulab Devi Hospital | 2016 | RIF, INH | AK, PYZ, LZD, MOX |
| MTBLH043 | Gulab Devi Hospital | 2016 | RIF, INH, AK, PYZ | LZD, MOX |
| MTBLH044 | Gulab Devi Hospital | 2016 | RIF, INH | AK, PYZ, LZD, MOX |
| MTBLH045 | Gulab Devi Hospital | 2016 | RIF, INH | AK, PYZ, LZD, MOX |
| MTBLH046 | Gulab Devi Hospital | 2016 | RIF, INH | AK, PYZ, LZD, MOX |
| MTBLH047 | Gulab Devi Hospital | 2016 | RIF, INH, PYZ | PYZ, LZD, MOX |
| MTBLH048 | Gulab Devi Hospital | 2016 | RIF, INH, PYZ, AK | LZD, MOX |
| MTBLH049 | Gulab Devi Hospital | 2016 | - | RIF, NIH, AK, PYZ, LZD, MOX |
| MTBLH050 | Gulab Devi Hospital | 2016 | RIF, INH | AK, PYZ, LZD, MOX |
| MTBLH051 | Gulab Devi Hospital | 2016 | RIF, INH, PYZ | AK, LZD, MOX |
| MTBLH052 | Gulab Devi Hospital | 2016 | RIF, INH | AK, PYZ, LZD, MOX |
| MTBLH053 | Gulab Devi Hospital | 2016 | RIF, INH, PYZ, AK | LZD, MOX |
| MTBLH054 | Gulab Devi Hospital | 2016 | RIF, INH, AK | PYZ, LZD, MOX |
| MTBLH055 | Gulab Devi Hospital | 2016 | RIF, INH, AK, PYZ | LZD, MOX |
| MTBLH056 | Gulab Devi Hospital | 2016 | RIF, INH | AK, PYZ, LZD, MOX |
| MTBLH057 | Mayo Hospital | 2016 | RIF, INH,PYZ | AK, MOX, LZD |
| MTBLH058 | Mayo Hospital | 2016 | RIF, INH, AK | PYZ, LZD, MOX |
| MTBLH059 | Mayo Hospital | 2016 | RIF, INH, AK, PYZ, LZD | MOX |
| MTBLH060 | Mayo Hospital | 2016 | RIF, INH, PYZ | AK, MOX, LZD |
| MTBLH061 | Mayo Hospital | 2016 | RIF, INH | AK, PYZ, MOX, LZD |
| MTBLH062 | Mayo Hospital | 2016 | RIF, INH, LZD | AK, PYZ, MOX |
| MTBLH063 | Mayo Hospital | 2016 | RIF, INH | AK, PYZ, LZD, MOX |
| MTBLH064 | Mayo Hospital | 2016 | RIF, INH | AK, PYZ, LZD, MOX |
| MTBLH065 | Mayo Hospital | 2016 | - | RIF, NIH, AK, PYZ, LZD, MOX |
| MTBLH066 | Mayo Hospital | 2016 | -- | RIF, NIH, AK, PYZ, LZD, MOX |
| MTBLH067 | Mayo Hospital | 2016 | - | RIF, NIH, AK, PYZ, LZD, MOX |
| MTBLH068 | Mayo Hospital | 2016 | - | RIF, NIH, AK, PYZ, LZD, MOX |
| MTBLH069 | Mayo Hospital | 2016 | AK | RIF, NIH, AK, PYZ, LZD, MOX |
| MTBLH070 | Mayo Hospital | 2016 | - | RIF, NIH, AK, PYZ, LZD, MOX |
| MTBLH071 | Mayo Hospital | 2016 | - | RIF, NIH, AK, PYZ, LZD, MOX |
| MTBLH072 | Mayo Hospital | 2016 | - | RIF, NIH, AK, PYZ, LZD, MOX |
| MTBLH073 | Mayo Hospital | 2016 | - | RIF, NIH, AK, PYZ, LZD, MOX |
| MTBLH074 | Mayo Hospital | 2016 |  | RIF, NIH, AK, PYZ, LZD, MOX |
| MTBLH075 | Mayo Hospital | 2016 |  | RIF, NIH, AK, PYZ, LZD, MOX |
| MTBLH076 | Mayo Hospital | 2016 |  | RIF, NIH, AK, PYZ, LZD, MOX |
| MTBLH077 | Mayo Hospital | 2016 |  | RIF, NIH, AK, PYZ, LZD, MOX |
| MTBLH078 | Mayo Hospital | 2017 |  | RIF, NIH, AK, PYZ, LZD, MOX |
| MTBLH079 | Mayo Hospital | 2017 |  | RIF, NIH, AK, PYZ, LZD, MOX |
| MTBLH080 | Mayo Hospital | 2017 |  | RIF, NIH, AK, PYZ, LZD, MOX |
| MTBLH081 | Mayo Hospital | 2017 |  | RIF, NIH, AK, PYZ, LZD, MOX |
| MTBLH082 | Mayo Hospital | 2017 |  | RIF, NIH, AK, PYZ, LZD, MOX |
| MTBLH083 | Mayo Hospital | 2017 |  | RIF, NIH, AK, PYZ, LZD, MOX |
| MTBLH084 | Mayo Hospital | 2017 |  | RIF, NIH, AK, PYZ, LZD, MOX |
| MTBLH085 | Mayo Hospital | 2017 | PYZ | RIF, NIH, AK, LZD, MOX |
| MTBLH086 | Gulab Devi Hospital | 2017 |  | RIF, NIH, AK, PYZ, LZD, MOX |
| MTBLH087 | Gulab Devi Hospital | 2017 |  | RIF, NIH, AK, PYZ, LZD, MOX |
| MTBLH088 | Gulab Devi Hospital | 2017 |  | RIF, NIH, AK, PYZ, LZD, MOX |
| MTBLH089 | Gulab Devi Hospital | 2017 |  | RIF, NIH, AK, PYZ, LZD, MOX |
| MTBLH090 | Gulab Devi Hospital | 2017 |  | RIF, NIH, AK, PYZ, LZD, MOX |
| MTBLH091 | Gulab Devi Hospital | 2017 |  | RIF, NIH, AK, PYZ, LZD, MOX |
| MTBLH092 | Gulab Devi Hospital | 2017 |  | RIF, NIH, AK, PYZ, LZD, MOX |
| MTBLH093 | Mayo Hospital | 2017 |  | RIF, NIH, AK, PYZ, LZD, MOX |
| MTBLH094 | Mayo Hospital | 2017 |  | RIF, NIH, AK, PYZ, LZD, MOX |
| MTBLH095 | Mayo Hospital | 2017 |  | RIF, NIH, AK, PYZ, LZD, MOX |
| MTBLH096 | Mayo Hospital | 2017 |  | RIF, NIH, AK, PYZ, LZD, MOX |
| MTBLH097 | Mayo Hospital | 2017 | PYZ | RIF, NIH, AK,LZD, MOX |
| MTBLH098 | Mayo Hospital | 2017 |  | RIF, NIH, AK, PYZ, LZD, MOX |
| MTBLH099 | Mayo Hospital | 2017 |  | RIF, NIH, AK, PYZ, LZD, MOX |
| MTBLH100 | Unique Lahore Lab | 2017 | RIF, NIH | AK, PYZ, LZD, MOX |
| MTBLH101 | Unique Lahore Lab | 2017 | RIF, NIH | AK, PYZ, LZD, MOX |
| MTBLH102 | Unique Lahore Lab | 2017 |  | RIF, NIH, AK, PYZ, LZD, MOX |
| MTBLH103 | Unique Lahore Lab | 2017 |  | RIF, NIH, AK, PYZ, LZD, MOX |
| MTBLH104 | Unique Lahore Lab | 2017 |  | RIF, NIH, AK, PYZ, LZD, MOX |
| MTBLH105 | Unique Lahore Lab | 2017 | RIF, NIH | AK, PYZ, LZD, MOX |
| MTBLH106 | Unique Lahore Lab | 2017 |  | RIF, NIH, AK, PYZ, LZD, MOX |
| MTBLH107 | Unique Lahore Lab | 2017 |  | RIF, NIH, AK, PYZ, LZD, MOX |
| MTBLH108 | Unique Lahore Lab | 2017 |  | RIF, NIH, AK, PYZ, LZD, MOX |
| MTBLH109 | Uniaque Lahore Lab | 2017 |  | RIF, NIH, AK, PYZ, LZD, MOX |
| MTBLH110 | Unique Lahore Lab | 2017 |  | RIF, NIH, AK, PYZ, LZD, MOX |
| MTBLH111 | Unique Lahore Lab | 2017 |  | RIF, NIH, AK, PYZ, LZD, MOX |
| MTBLH112 | Uniaque Lahore Lab | 2017 | PYZ | RIF, NIH, AK, LZD, MOX |
| MTBLH113 | Unique Lahore Lab | 2017 |  | RIF, NIH, AK, PYZ, LZD, MOX |
| MTBLH114 | Unique Lahore Lab | 2017 |  | RIF, NIH, AK, PYZ, LZD, MOX |
| MTBLH115 | Uniaque Lahore Lab | 2017 |  | RIF, NIH, AK, PYZ, LZD, MOX |
| MTBLH116 | Unique Lahore Lab | 2017 |  | RIF, NIH, AK, PYZ, LZD, MOX |
| MTBLH117 | Unique Lahore Lab | 2017 |  | RIF, NIH, AK, PYZ, LZD, MOX |
| MTBLH118 | Uniaque Lahore Lab | 2017 |  | RIF, NIH, AK, PYZ, LZD, MOX |
| MTBLH119 | Unique Lahore Lab | 2017 |  | RIF, NIH, AK, PYZ, LZD, MOX |
| MTBLH120 | Unique Lahore Lab | 2017 |  | RIF, NIH, AK, PYZ, LZD, MOX |
| MTBLH121 | Uniaque Lahore Lab | 2017 |  | RIF, NIH, AK, PYZ, LZD, MOX |
| MTBLH122 | Unique Lahore Lab | 2017 |  | RIF, NIH, AK, PYZ, LZD, MOX |
| MTBLH123 | Uniaque Lahore Lab | 2017 |  | RIF, NIH, AK, PYZ, LZD, MOX |
| MTBLH124 | Unique Lahore Lab | 2017 |  | RIF, NIH, AK, PYZ, LZD, MOX |
| MTBLH125 | Unique Lahore Lab | 2017 |  | RIF, NIH, AK, PYZ, LZD, MOX |
| MTBLH126 | Uniaque Lahore Lab | 2017 |  | RIF, NIH, AK, PYZ, LZD, MOX |
| MTBLH127 | Unique Lahore Lab | 2017 |  | RIF, NIH, AK, PYZ, LZD, MOX |
| MTBLH128 | Unique Lahore Lab | 2017 |  | RIF, NIH, AK, PYZ, LZD, MOX |

| **Strain ID** | **Place of isolation** | **Year of isolation** | **Drug resistance pattern** | **Drug ssusceptibility pattern** |
| --- | --- | --- | --- | --- |
| MTBLH001 | Mayo Hospital | 2016 | RIF, INH | AK, PYZ, LZD, MOX |
| MTBLH002 | Mayo Hospital | 2016 | AK | INH, RIF, PYZ, LZD, MOX |
| MTBLH003 | Mayo Hospital | 2016 | PYZ | INH, RIF, AK LZD, MOX |
| MTBLH004 | Mayo Hospital | 2016 | - | INH, RIF, AK, PYZ, LZD, MOX |
| MTBLH005 | Mayo Hospital | 2016 | . | INH, RIF, AK, PYZ, LZD, MOX |
| MTBLH006 | Mayo Hospital | 2016 | - | INH, RIF, AK, PYZ, LZD, MOX |
| MTBLH007 | Mayo Hospital | 2016 | RIF, INH | AK, PYZ, LZD, MOX |
| MTBLH008 | Mayo Hospital | 2016 | AK, PYZ | RIF, INH, LZD, MOX |
| MTBLH009 | Mayo Hospital | 2016 | - | INH, RIF, AK, PYZ, LZD, MOX |
| MTBLH010 | Mayo Hospital | 2016 | RIF, INH, AK | PYZ, LZD, MOX |
| MTBLH011 | Mayo Hospital | 2016 | - | INH, RIF, AK, PYZ, LZD, MOX |
| MTBLH012 | Mayo Hospital | 2016 | - | INH, RIF, AK, PYZ, LZD, MOX |
| MTBLH013 | Mayo Hospital | 2016 | PYZ | INH, RIF, AK, LZD, MOX |
| MTBLH014 | Mayo Hospital | 2016 | - | INH, RIF, AK, PYZ, LZD, MOX |
| MTBLH015 | Mayo Hospital | 2016 |  | INH, RIF, AK, PYZ, LZD, MOX |
| MTBLH016 | Mayo Hospital | 2016 | INH, RIF | AK, PYZ, LZD, MOX |
| MTBLH017 | Mayo Hospital | 2016 | PYZ | INH, RIF, AK, LZD, MOX |
| MTBLH018 | Mayo Hospital | 2016 | INH, RIF, AK, PYZ | LZD, MOX |
| MTBLH019 | Mayo Hospital | 2016 | - | INH, RIF, AK, PYZ, LZD, MOX |
| MTBLH020 | Gulab Devi Hospital | 2016 | PYZ | INH, RIF, AK, LZD, MOX |
| MTBLH021 | Gulab Devi Hospital | 2016 | RIF, INH,PYZ | AK, LZD, MOX |
| MTBLH022 | Gulab Devi Hospital | 2016 | RIF, INH,PYZ | AK, LZD, MOX |
| MTBLH023 | Gulab Devi Hospital | 2016 | RIF, INH, AK | PYZ, LZD, MOX |
| MTBLH024 | Gulab Devi Hospital | 2016 | RIF, INH | AK, PYZ, LZD, MOX |
| MTBLH025 | Gulab Devi Hospital | 2016 | RIF, INH,PYZ | AK, LZD, MOX |
| MTBLH026 | Gulab Devi Hospital | 2016 | RIF, INH,AK, PYZ | LZD, MOX |
| MTBLH027 | Gulab Devi Hospital | 2016 | RIF, INH,AK, PYZ | LZD, MOX |
| MTBLH028 | Gulab Devi Hospital | 2016 | RIF, INH,AK, PYZ | LZD, MOX |
| MTBLH029 | Gulab Devi Hospital | 2016 | RIF, INH, PYZ | AK, LZD, MOX |
| MTBLH030 | Gulab Devi Hospital | 2016 | RIF, INH, PYZ | AK, LZD, MOX |
| MTBLH031 | Gulab Devi Hospital | 2016 | RIF, INH,AK, PYZ | LZD, MOX |
| MTBLH032 | Gulab Devi Hospital | 2016 | RIF, INH, PYZ | AK, LZD, MOX |
| MTBLH033 | Gulab Devi Hospital | 2016 | RIF, INH, PYZ | AK, LZD, MOX |
| MTBLH034 | Gulab Devi Hospital | 2016 | RIF, INH, PYZ | AK, LZD, MOX |
| MTBLH035 | Gulab Devi Hospital | 2016 | RIF, INH | AK, PYZ, LZD, MOX |
| MTBLH036 | Gulab Devi Hospital | 2016 | - | RIF, INH, AK, LZD, MOX |
| MTBLH037 | Gulab Devi Hospital | 2016 | RIF, INH, PYZ | AK, LZD, MOX |
| MTBLH038 | Gulab Devi Hospital | 2016 | RIF, INH, AK, PYZ | MOX, LZD |
| MTBLH039 | Gulab Devi Hospital | 2016 | RIF, INH | AK, PYZ, LZD, MOX |
| MTBLH040 | Gulab Devi Hospital | 2016 | RIF, INH, PYZ | AK, PYZ, LZD, MOX |
| MTBLH041 | Gulab Devi Hospital | 2016 | RIF, INH | AK, PYZ, LZD, MOX |
| MTBLH042 | Gulab Devi Hospital | 2016 | RIF, INH | AK, PYZ, LZD, MOX |
| MTBLH043 | Gulab Devi Hospital | 2016 | RIF, INH, AK, PYZ | LZD, MOX |
| MTBLH044 | Gulab Devi Hospital | 2016 | RIF, INH | AK, PYZ, LZD, MOX |
| MTBLH045 | Gulab Devi Hospital | 2016 | RIF, INH | AK, PYZ, LZD, MOX |
| MTBLH046 | Gulab Devi Hospital | 2016 | RIF, INH | AK, PYZ, LZD, MOX |
| MTBLH047 | Gulab Devi Hospital | 2016 | RIF, INH, PYZ | PYZ, LZD, MOX |
| MTBLH048 | Gulab Devi Hospital | 2016 | RIF, INH, PYZ, AK | LZD, MOX |
| MTBLH049 | Gulab Devi Hospital | 2016 | - | RIF, NIH, AK, PYZ, LZD, MOX |
| MTBLH050 | Gulab Devi Hospital | 2016 | RIF, INH | AK, PYZ, LZD, MOX |
| MTBLH051 | Gulab Devi Hospital | 2016 | RIF, INH, PYZ | AK, LZD, MOX |
| MTBLH052 | Gulab Devi Hospital | 2016 | RIF, INH | AK, PYZ, LZD, MOX |
| MTBLH053 | Gulab Devi Hospital | 2016 | RIF, INH, PYZ, AK | LZD, MOX |
| MTBLH054 | Gulab Devi Hospital | 2016 | RIF, INH, AK | PYZ, LZD, MOX |
| MTBLH055 | Gulab Devi Hospital | 2016 | RIF, INH, AK, PYZ | LZD, MOX |
| MTBLH056 | Gulab Devi Hospital | 2016 | RIF, INH | AK, PYZ, LZD, MOX |
| MTBLH057 | Mayo Hospital | 2016 | RIF, INH,PYZ | AK, MOX, LZD |
| MTBLH058 | Mayo Hospital | 2016 | RIF, INH, AK | PYZ, LZD, MOX |
| MTBLH059 | Mayo Hospital | 2016 | RIF, INH, AK, PYZ, LZD | MOX |
| MTBLH060 | Mayo Hospital | 2016 | RIF, INH, PYZ | AK, MOX, LZD |
| MTBLH061 | Mayo Hospital | 2016 | RIF, INH | AK, PYZ, MOX, LZD |
| MTBLH062 | Mayo Hospital | 2016 | RIF, INH, LZD | AK, PYZ, MOX |
| MTBLH063 | Mayo Hospital | 2016 | RIF, INH | AK, PYZ, LZD, MOX |
| MTBLH064 | Mayo Hospital | 2016 | RIF, INH | AK, PYZ, LZD, MOX |
| MTBLH065 | Mayo Hospital | 2016 | - | RIF, NIH, AK, PYZ, LZD, MOX |
| MTBLH066 | Mayo Hospital | 2016 | -- | RIF, NIH, AK, PYZ, LZD, MOX |
| MTBLH067 | Mayo Hospital | 2016 | - | RIF, NIH, AK, PYZ, LZD, MOX |
| MTBLH068 | Mayo Hospital | 2016 | - | RIF, NIH, AK, PYZ, LZD, MOX |
| MTBLH069 | Mayo Hospital | 2016 | AK | RIF, NIH, AK, PYZ, LZD, MOX |
| MTBLH070 | Mayo Hospital | 2016 | - | RIF, NIH, AK, PYZ, LZD, MOX |
| MTBLH071 | Mayo Hospital | 2016 | - | RIF, NIH, AK, PYZ, LZD, MOX |
| MTBLH072 | Mayo Hospital | 2016 | - | RIF, NIH, AK, PYZ, LZD, MOX |
| MTBLH073 | Mayo Hospital | 2016 | - | RIF, NIH, AK, PYZ, LZD, MOX |
| MTBLH074 | Mayo Hospital | 2016 |  | RIF, NIH, AK, PYZ, LZD, MOX |
| MTBLH075 | Mayo Hospital | 2016 |  | RIF, NIH, AK, PYZ, LZD, MOX |
| MTBLH076 | Mayo Hospital | 2016 |  | RIF, NIH, AK, PYZ, LZD, MOX |
| MTBLH077 | Mayo Hospital | 2016 |  | RIF, NIH, AK, PYZ, LZD, MOX |
| MTBLH078 | Mayo Hospital | 2017 |  | RIF, NIH, AK, PYZ, LZD, MOX |
| MTBLH079 | Mayo Hospital | 2017 |  | RIF, NIH, AK, PYZ, LZD, MOX |
| MTBLH080 | Mayo Hospital | 2017 |  | RIF, NIH, AK, PYZ, LZD, MOX |
| MTBLH081 | Mayo Hospital | 2017 |  | RIF, NIH, AK, PYZ, LZD, MOX |
| MTBLH082 | Mayo Hospital | 2017 |  | RIF, NIH, AK, PYZ, LZD, MOX |
| MTBLH083 | Mayo Hospital | 2017 |  | RIF, NIH, AK, PYZ, LZD, MOX |
| MTBLH084 | Mayo Hospital | 2017 |  | RIF, NIH, AK, PYZ, LZD, MOX |
| MTBLH085 | Mayo Hospital | 2017 | PYZ | RIF, NIH, AK, LZD, MOX |
| MTBLH086 | Gulab Devi Hospital | 2017 |  | RIF, NIH, AK, PYZ, LZD, MOX |
| MTBLH087 | Gulab Devi Hospital | 2017 |  | RIF, NIH, AK, PYZ, LZD, MOX |
| MTBLH088 | Gulab Devi Hospital | 2017 |  | RIF, NIH, AK, PYZ, LZD, MOX |
| MTBLH089 | Gulab Devi Hospital | 2017 |  | RIF, NIH, AK, PYZ, LZD, MOX |
| MTBLH090 | Gulab Devi Hospital | 2017 |  | RIF, NIH, AK, PYZ, LZD, MOX |
| MTBLH091 | Gulab Devi Hospital | 2017 |  | RIF, NIH, AK, PYZ, LZD, MOX |
| MTBLH092 | Gulab Devi Hospital | 2017 |  | RIF, NIH, AK, PYZ, LZD, MOX |
| MTBLH093 | Mayo Hospital | 2017 |  | RIF, NIH, AK, PYZ, LZD, MOX |
| MTBLH094 | Mayo Hospital | 2017 |  | RIF, NIH, AK, PYZ, LZD, MOX |
| MTBLH095 | Mayo Hospital | 2017 |  | RIF, NIH, AK, PYZ, LZD, MOX |
| MTBLH096 | Mayo Hospital | 2017 |  | RIF, NIH, AK, PYZ, LZD, MOX |
| MTBLH097 | Mayo Hospital | 2017 | PYZ | RIF, NIH, AK,LZD, MOX |
| MTBLH098 | Mayo Hospital | 2017 |  | RIF, NIH, AK, PYZ, LZD, MOX |
| MTBLH099 | Mayo Hospital | 2017 |  | RIF, NIH, AK, PYZ, LZD, MOX |
| MTBLH100 | Unique Lahore Lab | 2017 | RIF, NIH | AK, PYZ, LZD, MOX |
| MTBLH101 | Unique Lahore Lab | 2017 | RIF, NIH | AK, PYZ, LZD, MOX |
| MTBLH102 | Unique Lahore Lab | 2017 |  | RIF, NIH, AK, PYZ, LZD, MOX |
| MTBLH103 | Unique Lahore Lab | 2017 |  | RIF, NIH, AK, PYZ, LZD, MOX |
| MTBLH104 | Unique Lahore Lab | 2017 |  | RIF, NIH, AK, PYZ, LZD, MOX |
| MTBLH105 | Unique Lahore Lab | 2017 | RIF, NIH | AK, PYZ, LZD, MOX |
| MTBLH106 | Unique Lahore Lab | 2017 |  | RIF, NIH, AK, PYZ, LZD, MOX |
| MTBLH107 | Unique Lahore Lab | 2017 |  | RIF, NIH, AK, PYZ, LZD, MOX |
| MTBLH108 | Unique Lahore Lab | 2017 |  | RIF, NIH, AK, PYZ, LZD, MOX |
| MTBLH109 | Uniaque Lahore Lab | 2017 |  | RIF, NIH, AK, PYZ, LZD, MOX |
| MTBLH110 | Unique Lahore Lab | 2017 |  | RIF, NIH, AK, PYZ, LZD, MOX |
| MTBLH111 | Unique Lahore Lab | 2017 |  | RIF, NIH, AK, PYZ, LZD, MOX |
| MTBLH112 | Uniaque Lahore Lab | 2017 | PYZ | RIF, NIH, AK, LZD, MOX |
| MTBLH113 | Unique Lahore Lab | 2017 |  | RIF, NIH, AK, PYZ, LZD, MOX |
| MTBLH114 | Unique Lahore Lab | 2017 |  | RIF, NIH, AK, PYZ, LZD, MOX |
| MTBLH115 | Uniaque Lahore Lab | 2017 |  | RIF, NIH, AK, PYZ, LZD, MOX |
| MTBLH116 | Unique Lahore Lab | 2017 |  | RIF, NIH, AK, PYZ, LZD, MOX |
| MTBLH117 | Unique Lahore Lab | 2017 |  | RIF, NIH, AK, PYZ, LZD, MOX |
| MTBLH118 | Uniaque Lahore Lab | 2017 |  | RIF, NIH, AK, PYZ, LZD, MOX |
| MTBLH119 | Unique Lahore Lab | 2017 |  | RIF, NIH, AK, PYZ, LZD, MOX |
| MTBLH120 | Unique Lahore Lab | 2017 |  | RIF, NIH, AK, PYZ, LZD, MOX |
| MTBLH121 | Uniaque Lahore Lab | 2017 |  | RIF, NIH, AK, PYZ, LZD, MOX |
| MTBLH122 | Unique Lahore Lab | 2017 |  | RIF, NIH, AK, PYZ, LZD, MOX |
| MTBLH123 | Uniaque Lahore Lab | 2017 |  | RIF, NIH, AK, PYZ, LZD, MOX |
| MTBLH124 | Unique Lahore Lab | 2017 |  | RIF, NIH, AK, PYZ, LZD, MOX |
| MTBLH125 | Unique Lahore Lab | 2017 |  | RIF, NIH, AK, PYZ, LZD, MOX |
| MTBLH126 | Uniaque Lahore Lab | 2017 |  | RIF, NIH, AK, PYZ, LZD, MOX |
| MTBLH127 | Unique Lahore Lab | 2017 |  | RIF, NIH, AK, PYZ, LZD, MOX |
| MTBLH128 | Unique Lahore Lab | 2017 |  | RIF, NIH, AK, PYZ, LZD, MOX |

**Table S4: Distribution of *Mycobacterium tuberculosis* isolates from treatment failure cases based on drug resistance**

| Non-drug resistant  Total =12 | Pyrazinamide (PAZ) Mono resistance  Total=3 | Amikacin (AK)  Mono resistance  Total=1 | Rifampicin + Isoniazid (MDR)  Total=17 | MDR+ PAZ  Total=15 | MDR+AK  (MDR)  Total=4 | MDR+AK+PAZ  (MDR)  Total=12 |
| --- | --- | --- | --- | --- | --- | --- |
|  |  |  | MDR =32 | | MDR=16 | |
| MTBLH004  MTBLH005  MTBLH006  MTBLH009  MTBLH011  MTBLH012  MTBLH014  MTBLH015  MTBLH016  MTBLH019  MTBLH036  MTBLH049 | MTBLH003  MTBLH013  MTBLH017 | MTBLH002 | MTBLH001  MTBLH007  MTBLH024  MTBLH035  MTBLH039  MTBLH041  MTBLH042  MTBLH044  MTBLH045  MTBLH046  MTBLH050  MTBLH052  MTBLH056  MTBLH061  MTBLH063  MTBLH064 | MTBLH020  MTBLH021  MTBLH022  MTBLH025  MTBLH029  MTBLH030  MTBLH032  MTBLH033  MTBLH034  MTBLH037  MTBLH040  MTBLH047  MTBLH051  MTBLH057  MTBLH060 | MTBLH010  MTBLH023  MTBLH054  MTBLH058 | MTBLH018  MTBLH026  MTBLH027  MTBLH028  MTBLH031  MTBLH038  MTBLH043  MTBLH048  MTBLH053  MTBLH055  MTBLH059  MTBLH062 |

**Table S5: Distribution of *Mycobacterium tuberculosis* resistance tuberculosis isolates from freshly diagnosed cases based on drug resistance**

| Non- drug resistant  Total =57 | Pyrazinamide (PAZ) Mono drug resistant  Total=3 | Amikacin (AK)  Mono drug resistant  Total=1 | Rifampicin+  Isoniazid (MDR)  Total=3 | MDR+PAZ  Total=0 | MDR+AK  (MDR)  Total=0 | MDR+AK+PAZ  (MDR)  Total=0 |
| --- | --- | --- | --- | --- | --- | --- |
| MTBLH065, MTBLH066, MTBLH067, MTBLH068, MTBLH070, MTBLH074  MTBLH071, MTBLH072, MTBLH073, MTBLH0 MTBLH075, MTBLH076, MTBLH077, MT B MTBLH078, MTBLH079, MTBLH080,  MTBLH081, MTBLH082, MTBLH083, MTBLH084, MTBLH086, MTBLH087, MTBLH088, MTBLH089, MTBLH090, MTBLH091, MTBLH092, MTBLH093, MTBLH094, MTBLH095, MTBLH096,  MTBLH098, MTBLH099, MTBLH102, MTBLH103, MTBLH104, MTBLH106, MTBLH107, MTBLH108, MTBLH109, MTBLH110, MTBLH111, MTBLH113, MTBLH114, MTBLH115, MTBLH116,  MTBLH117, MTBLH118, MTBLH119, MTBLH120, MTBLH121, MTBLH122, MTBLH123, MTBLH124, MTBLH125, MTBLH126, MTBLH127, MTBLH128 | MTBLH085,  MTBLH097,  MTBLH112, | MTBLH069 | MTBLH100  MTBLH101  MTBLH105 |  |  |  |

**Table S6: Sensitivity pattern of Rifampicin resistant isolates at different concentrations of verapamil in the presence and absence of Rifampicin**

| **No** | ***M. tuberculosis* isolate ID/Phenotype**  **MDR/MDR** | **0 µg/ml VP** | | **5 µg/ml VP** | | **64 µg/ml VP** | | **128 µg/ml VP** | | **256 µg/ml VP** | | **512 µg/ml VP** | |
| --- | --- | --- | --- | --- | --- | --- | --- | --- | --- | --- | --- | --- | --- |
|  |  | **0 µg Rif** | **1.0 µg Rif** | **0 µg Rif** | **1.0 µg Rif** | **0 µg Rif** | **1.0 µg Rif** | **0 µg Rif** | **1.0 µg Rif** | **0 µg Rif** | **1.0 µg Rif** | **0 µg Rif** | **1.0 µg Rif** |
| 1 | MTBLH001 | + | + | + | + | + | + | + | + | _ | _ | _ | _ |
| 2 | MTBLH007 | + | + | + | + | + | + | + | + | _ | _ | _ | _ |
| 3 | MTBLH 010 | + | + | + | + | + | + | + | + | _ | _ | _ | _ |
| 4 | MTBLH021 | + | + | + | + | + | + | + | + | _ | _ | _ | _ |
| 5 | MTBLH022 | + | + | + | + | + | + | + | + | _ | _ | _ | _ |
| 6 | MTBLH 023 | + | + | + | + | + | + | + | + | _ | _ | _ | _ |
| 7 | MTBLH025 | + | + | + | + | + | + | + | + | _ | _ | _ | _ |
| 8 | MTBLH026 | + | + | + | + | + | + | + | + | _ | _ | _ | _ |
| 9 | MTBLH 027 | + | + | + | + | + | + | + | + | _ | _ | _ | _ |
| 10 | MTBLH 028 | + | + | + | + | + | + | + | + | + | _ | _ | _ |
| 11 | MTBLH 029 | + | + | + | + | + | + | + | + | _ | _ | _ | _ |
| 12 | MTBLH 016 | + | + | + | + | + | + | + | + | _ | _ | _ | _ |
| 13 | MTBLH 018 | + | + | + | + | + | + | + | + | + | _ | _ | _ |
| 14 | MTBLH 059 | + | + | + | + | + | + | + | + | + | _ | _ | _ |
| 15 | MTBLH 062 | + | + | + | + | + | + | + | + | _ | _ | _ | _ |
| 16 | MTBLH060 | + | + | + | + | + | + | + | + | _ | _ | _ | _ |
| 17 | MTBLH053 | + | + | + | + | + | + | + | + | + | _ | _ | _ |
| 18 | MTBLH 032 | + | + | + | + | + | + | + | + | _ | _ | _ | _ |
| 19 | MTBLH 033 | + | + | + | + | + | + | + | + | _ | _ | _ | _ |
| 20 | MTBLH 034 | + | + | + | + | + | + | + | + | _ | _ | _ | _ |
| 21 | MTBLH 035 | + | + | + | + | + | + | + | + | _ | _ | _ | _ |
| 22 | MTBLH 037 | + | + | + | + | + | + | + | + | _ | _ | _ | _ |
| 23 | MTBLH 038 | + | + | + | + | + | + | + | + | _ | _ | _ | _ |
| 24 | MTBLH 039 | + | + | + | + | + | + | + | + | _ | _ | _ | _ |
| 25 | MTBLH 040 | + | + | + | + | + | + | + | + | _ | _ | _ | _ |
| 26 | MTBLH 041 | + | + | + | + | + | + | + | + | _ | _ | _ | _ |
| 27 | MTBLH 042 | + | + | + | + | + | + | + | + | _ | _ | _ | _ |
| 28 | MDR- MTBLH 043 | + | + | + | + | + | + | + | + | _ | _ | _ | _ |
| 29 | MTBLH 044 | + | + | + | + | + | + | + | + | _ | _ | _ | _ |
| 30 | MTBLH045 | + | + | + | + | + | + | + | + | _ | _ | _ | _ |
| 31 | MTBLH 046 | + | + | + | + | + | + | + | + | _ | _ | _ | _ |
| 32 | MTBLH 047 | + | + | + | + | + | + | + | + | _ | _ | _ | _ |
| 33 | MTBLH 048 | + | + | + | + | + | + | + | + | _ | _ | _ | _ |

+ = Growth (Resistant) - **= No Growth (Susceptible)**

**Table S7: Sensitivity pattern of Isoniazid resistant isolates at different concentrations of Verapamil in the presence and absence of Isoniazid.**

| **No** | ***M. tuberculosis* isolate ID/Phenotype** | **0 µg/ml VP** | | **5 µg/ml VP** | | **64 µg/ml VP** | | **128 µg/ml VP** | | **256 µg/ml VP** | | **512 µg/ml VP** | |
| --- | --- | --- | --- | --- | --- | --- | --- | --- | --- | --- | --- | --- | --- |
|  |  | **0 µg/ml INH** | **0.1 µg/ml INH** | **0 µg/ml INH** | **0.1 µg/ml INH** | **0 µg/ml INH** | **0.1 µg/ml INH** | **0 µg/ml INH** | **0.1 µg/ml INH** | **0 µg/ml INH** | **0.1 µg/ml INH** | **0 µg/ml INH** | **0.1 µg/ ml INH** |
| 1 | MTBLH001 | + | + | + | + | + | + | + | + | _ | _ | _ | _ |
| 2 | MTBLH007 | + | + | + | + | + | + | + | + | _ | _ | _ | _ |
| 3 | MTBLH 010 | + | + | + | + | + | + | + | + | _ | _ | _ | _ |
| 4 | MTBLH021 | + | + | + | + | + | + | + | + | _ | _ | _ | _ |
| 5 | MTBLH022 | + | + | + | + | + | + | + | + | _ | _ | _ | _ |
| 6 | MTBLH 023 | + | + | + | + | + | + | + | + | _ | _ | _ | _ |
| 7 | MTBLH 025 | + | + | + | + | + | + | + | + | _ | _ | _ | _ |
| 8 | MTBLH 026 | + | + | + | + | + | + | + | + | _ | _ | _ | _ |
| 9 | MTBLH 027 | + | + | + | + | + | + | + | + | _ | _ | _ | _ |
| 10 | MTBLH 028 | + | + | + | + | + | + | + | + | + | _ | _ | _ |
| 11 | MTBLH 029 | + | + | + | + | + | + | + | + | _ | _ | _ | _ |
| 12 | MTBLH 016 | + | + | + | + | + | + | + | + | _ | _ | _ | _ |
| 13 | MTBLH 018 | + | + | + | + | + | + | + | + | + | _ | _ | _ |
| 14 | MTBLH 059 | + | + | + | + | + | + | + | + | + | _ | _ | _ |
| 15 | MTBLH 062 | + | + | + | + | + | + | + | + | _ | _ | _ | _ |
| 16 | MTBLH 060 | + | + | + | + | + | + | + | + | _ | _ | _ | _ |
| 17 | MTBLH 053 | + | + | + | + | + | + | + | + | + | _ | _ | _ |
| 18 | MTBLH 032 | + | + | + | + | + | + | + | + | _ | _ | _ | _ |
| 19 | MTBLH 033 | + | + | + | + | + | + | + | + | _ | _ | _ | _ |
| 20 | MTBLH 034 | + | + | + | + | + | + | + | + | _ | _ | _ | _ |
| 21 | MTBLH 035 | + | + | + | + | + | + | + | + | _ | _ | _ | _ |
| 22 | MTBLH 037 | + | + | + | + | + | + | + | + | _ | _ | _ | _ |
| 23 | MTBLH 038 | + | + | + | + | + | + | + | + | _ | _ | _ | _ |
| 24 | MTBLH 039 | + | + | + | + | + | + | + | + | _ | _ | _ | _ |
| 25 | MTBLH 040 | + | + | + | + | + | + | + | + | _ | _ | _ | _ |
| 26 | MTBLH 040 | + | + | + | + | + | + | + | + | _ | _ | _ | _ |
| 27 | MTBLH 042 | + | + | + | + | + | + | + | + | _ | _ | _ | _ |
| 28 | MTBLH 043 | + | + | + | + | + | + | + | + | _ | _ | _ | _ |
| 29 | MTBLH 044 | + | + | + | + | + | + | + | + | _ | _ | _ | _ |
| 30 | MTBLH045 | + | + | + | + | + | + | + | + | _ | _ | _ | _ |
| 31 | MTBLH 046 | + | + | + | + | + | + | + | + | _ | _ | _ | _ |
| 32 | TBLH 047 | + | + | + | + | + | + | + | + | _ | _ | _ | _ |
| 33 | MTBLH 048 | + | + | + | + | + | + | + | + | _ | _ | _ | _ |

+ = Growth (Resistant) - **= No Growth (Susceptible)**

**Table S8: Sensitivity pattern of Amikacin resistant isolates at different concentrations of Verapamil in the presence and absence of Amikacin**

| **No** | **Isolate ID**  **ID** | **0 µg/ml VP** | | **5 µg/ml VP** | | **64µg/ml VP** | | **128µg/ml VP** | | **256µg/ml VP** | | **512µg/ml VP** | |
| --- | --- | --- | --- | --- | --- | --- | --- | --- | --- | --- | --- | --- | --- |
|  |  | **0 µg AK** | **1.0µg AK** | **0µg AK** | **1.0µg AK** | **0 µg AK** | **1.0µg AK** | **0 µg AK** | **1.0µg AK** | **0µg AK** | **1.0µg AK** | **0µg AK** | **1.0µg AK** |
| 1 | MTBLH002 | + | + | + | + | + | + | + | + | _ | _ | _ | _ |
| 2 | MTBLH008 | + | + | + | + | + | + | + | + | _ | _ | _ | _ |
| 3 | MTBLH010 | + | + | + | + | + | + | + | + | _ | _ | _ | _ |
| 4 | MTBLH018 | + | + | + | + | + | + | + | + | _ | _ | _ | _ |
| 5 | MTBLH023 | + | + | + | + | + | + | + | + | _ | _ | _ | _ |
| 6 | MTBLH026 | + | + | + | + | + | + | + | + | _ | _ | _ | _ |
| 7 | MTBLH028 | + | + | + | + | + | + | + | + | + | + | _ | _ |
| 8 | MTBLH018 | + | + | + | + | + | + | + | + | + | + | _ | _ |
| 9 | MTBLH059 | + | + | + | + | + | + | + | + | + | + | _ | _ |
| 10 | MTBLH053 | + | + | + | + | + | + | + | + | + | + | _ | _ |
| 11 | MTBLH058 | + | + | + | + | + | + | + | + | _ | _ | _ | _ |
| 12 | MTBLH043 | + | + | + | + | + | + | + | + | _ | _ | _ | _ |
| 13 | MTBLH048 | + | + | + | + | + | + | + | + | _ | _ | _ | _ |

1 Chae, H. *et al.* Development of a One-Step Multiplex PCR Assay for Differential Detection of Major Mycobacterium Species. *J Clin Microbiol* **55**, 2736-2751, doi:10.1128/jcm.00549-17 (2017).

Reference
