# Supplementary material for: Antimicrobial drug resistant features of Mycobacterium tuberculosis associated with treatment failure
Source: PLoS One. 2023 Oct 26;18(10):e0293194. doi: 10.1371/journal.pone.0293194 (PMC10602240; doi:10.1371/journal.pone.0293194)
Supplement: Attachment — Submitted filename: rebutal letter.docx [file pone.0293194.s004.docx]

Reviewer #2: The authors are advised to recheck the manuscript for typographical errors as they are still present throughout the manuscript. All standard tests should be quoted appropriately eg. Gene XPert MTB/RIF is written as Gene Expert through out; figure titles 'resistant' is spelled as ressitant . There are many spelling errors which should be corrected.

*Authors´ reply:*

We are thankful to the reviewer for bringing the attention to typographical errors. We have now fully proofread the manuscript to remove such errors.

2. After revising the manuscript the Discussion portion does not seem homogenous. It should be rewritten.

*Authors´ reply:*

We agree with reviewer that the discussion was not homogenous. We have now rewritten the discussion to make it more coherent and homogeneous.
